# Web-Based Cognitive Testing in Psychiatric Research: Validation and Usability Study

**DOI:** 10.2196/28233

**Published:** 2022-02-10

**Authors:** Amy Joanne Lynham, Ian R Jones, James T R Walters

**Affiliations:** 1 Medical Research Council Centre for Neuropsychiatric Genetics and Genomics Division of Psychiatry and Clinical Neurosciences, School of Medicine Cardiff University Cardiff United Kingdom

**Keywords:** cognition, mental health, online, digital, assessment, validation, memory, attention, mobile phone

## Abstract

**Background:**

Cognitive impairments are features of many psychiatric disorders and affect functioning. A barrier to cognitive research on psychiatric disorders is the lack of large cross-disorder data sets. However, the collection of cognitive data can be logistically challenging and expensive. Web-based collection may be an alternative; however, little is known about who does and does not complete web-based cognitive assessments for psychiatric research.

**Objective:**

The aims of this study are to develop a web-based cognitive battery for use in psychiatric research, validate the battery against the Measurement and Treatment Research to Improve Cognition in Schizophrenia (MATRICS) Consensus Cognitive Battery, and compare the characteristics of the participants who chose to take part with those of the individuals who did not participate.

**Methods:**

Tasks were developed by The Many Brains Project and selected to measure the domains specified by the MATRICS initiative. We undertook a cross-validation study of 65 participants with schizophrenia, bipolar disorder, depression, or no history of psychiatric disorders to compare the web-based tasks with the MATRICS Consensus Cognitive Battery. Following validation, we invited participants from 2 large ongoing genetic studies, which recruited participants with psychiatric disorders to complete the battery and evaluated the demographic and clinical characteristics of those who took part.

**Results:**

Correlations between web-based and MATRICS tasks ranged between 0.26 and 0.73. Of the 961 participants, 887 (92.3%) completed at least one web-based task, and 644 (67%) completed all tasks, indicating adequate completion rates. Predictors of web-based participation included being female (odds ratio [OR] 1.3, 95% CI 1.07-1.58), ethnicity other than White European (OR 0.66, 95% CI 0.46-0.96), higher levels of education (OR 1.19, 95% CI 1.11-1.29), diagnosis of an eating disorder (OR 2.17, 95% CI 1.17-4) or depression and anxiety (OR 5.12, 95% CI 3.38-7.83), and absence of a diagnosis of schizophrenia (OR 0.59, 95% CI 0.35-0.94). Lower performance on the battery was associated with poorer functioning (B=−1.76, SE 0.26; *P*<.001).

**Conclusions:**

Our findings offer valuable insights into the advantages and disadvantages of testing cognitive function remotely for mental health research.

## Introduction

### Background

Cognitive impairments are core features of many psychiatric disorders [1-3], persist during remission [2,3], are not alleviated by current treatments [4-6], and are associated with poor functional outcomes [7,8]. Existing cognitive research on psychiatric disorders is limited by relatively small sample sizes, as collecting cognitive data can be labor intensive, and the use of existing cognitive batteries can be expensive given licensing restrictions. A potential solution to this is to use web-based data collection methods, which may be an effective way of acquiring large amounts of cognitive data using minimal resources. Data can be collected remotely using specially designed tasks that are administered via the internet and accessed using the participants’ own devices and web browsers in an unsupervised setting, such as the participants’ homes. The advantages of web-based methods include (1) relatively inexpensive costs per participant [9,10], (2) automatic data entry that limits errors [10], (3) ability to recruit from locations that would normally be out of reach [11], and (4) promotion of research to the public [11].

To date, studies comparing web-based and laboratory-based cognitive tasks have reported high correlations, few systematic differences between the assessments, and good internal reliability of web-based tasks [9,12-15]. One such study is the global citizen science project, *TestMyBrain*, which has collected cognitive data via the internet on >2 million volunteers around the world. Analysis of the data collected from the website compared with data collected using the same tasks under supervision in a research laboratory showed few differences in mean performance, variance, and internal reliability between the 2 sets of tasks [12]. A recent study of UK Biobank (UKB) participants demonstrated adequate concurrent validity between 11 cognitive assessments administered without supervision and 11 previously validated tests from the published literature (correlations between 0.22 and 0.83) [16]. The highest correlation (*r*=0.83) was observed between versions of the same task—the National Institutes of Health Toolbox Picture Vocabulary Test—whereas the lowest correlations were observed between tasks with differing methodologies (UKB Pairs Matching test and Wechsler Memory Scale-IV Designs Total; *r*=−0.33) or tasks with low levels of performance variance (UKB Prospective Memory test and Rivermead Behavioral Memory Test Appointments; *r*=0.22). Similar results were reported in a study comparing the Amsterdam Cognition Scan with a traditional test battery (correlations ranged between 0.36 and 0.78), where lower correlations were observed in tasks that had differing designs [15]. Another study compared identical Cambridge Neuropsychological Test Automated Battery tasks administered unsupervised via the internet and administered in person at a research facility (*r*=0.39-0.73) [14]. Correlations were similar to previously reported test–retest reliabilities for the tasks, although it was noted that tasks with a reaction time component were less comparable across the different administration settings.

To date, studies have evaluated the use of web-based cognitive assessments in healthy population samples. Therefore, questions remain about whether web-based tasks conducted in an unsupervised setting without a researcher present are suitable for research on individuals with psychiatric disorders. This is a particularly important question, given that these individuals are more likely to have moderate to severe cognitive impairments. An issue is participation bias, as web-based studies may exclude individuals who are less computer literate or who do not have internet access, such as older adults, those with lower incomes or education levels, or those with more severe psychiatric disorders. For example, a study that used Facebook to recruit participants for a mental health survey found that the participants were younger, more likely to be female, more educated, and more likely to be English speakers compared with the national averages taken from census data and a population study [17].

### Objectives

This study has 2 aims. Our first aim is to develop and validate a web-based cognitive battery for use in psychiatric research. Our second aim is to determine whether web-based cognitive testing is a suitable method for large-scale mental health research. By offering participation in a web-based cognitive study to those who had already been recruited into a cohort of individuals with mental health conditions, we will be able to identify the characteristics of those who chose to take part and compare them with those who did not. This study is presented in two parts: (1) validation of the web-based battery and (2) expansion of the web-based battery via recruitment of a large cross-disorder sample.

## Methods

### Participants

Participants from 2 studies conducted within the Medical Research Council Centre for Neuropsychiatric Genetics and Genomics at Cardiff University were invited to take part: (1) the National Centre for Mental Health (NCMH) cohort study [18] and (2) the Cognition in Mood, Psychosis, and Schizophrenia Study (CoMPaSS) [19]. Diagnoses were ascertained through either self-report in the NCMH or through a clinical interview in the CoMPaSS (Schedule for Clinical Assessment in Neuropsychiatry [20]). In the NCMH, participants were asked the following question: “Has a doctor or health professional ever told you that you have any of the following diagnoses?” Participants were given a list of diagnoses and asked to indicate all diagnoses that applied, the diagnosis they considered to be their primary diagnosis, and whether their clinical team would agree. Both studies included confirmation of consent from participants to be approached for other research within the Centre and consent for medical records to be accessed to obtain information regarding diagnosis and other clinical details. The full details of these studies can be found in Multimedia Appendix 1 [12,19-39]. All stages of the web-based study received ethical approval from the School of Medicine research ethics committee at Cardiff University (reference number 15/64), and each parent study had National Health Service ethical approval (NCMH reference number: 16/WA/0323; CoMPaSS reference number: 07/WSE03/110). The participants indicated their consent on the web by ticking a box at the bottom of the information page. We did not collect any identifiable information about the participants during the assessments; the participants’ data were pseudonymized and linked with an ID number. The assessment websites complied with current UK data security best practice guidelines in consultation with the Information Technology Systems Security Team and Research Governance Officers at Cardiff University.

### Neuropsychological Assessments

#### Web-Based Battery

All tasks were selected from and hosted on The Many Brains Project’s web-based cognitive testing platform, TestMyBrain [12,40]. We selected the tasks to assess, as closely as possible, the domains outlined by the National Institute of Mental Health–Measurement and Treatment Research to Improve Cognition in Schizophrenia (MATRICS) initiative [41]. We were able to include a formally equivalent web-based version of digit symbol coding. However, it was not possible to select formally equivalent tasks for the remaining domains because of a lack of availability of web-based versions. In addition to these domains, we included a measure of crystallized intelligence and a measure of risk-taking propensity. The final battery included 9 tasks (see Table 1 for domains, tasks, and equivalent MATRICS Consensus Cognitive Battery [MCCB] tasks). Full descriptions of the tasks can be found in Multimedia Appendix 1. The total administration time was approximately 45 to 50 minutes.

The tests were designed to run on desktop and laptop computers, touchscreen tablet computers, and smartphones. The Many Brains Project developed the cognitive tasks and hosted them on their secure TestMyBrain server, which could be accessed using a study-specific website link. The assessments were loaded in the participant’s internet browser, and the data were stored locally during each task. At the end of each task, the data were encrypted and uploaded to a secure server.

**Table 1:** Web-based and Measurement and Treatment Research to Improve Cognition in Schizophrenia Consensus Cognitive Battery (MCCB) tasks^a^.

Domain	MCCB task	Web-based task
Speed of processing	BACS^b^: digit symbol coding	Digit symbol coding
Social cognition	MSCEIT^c^: Managing Emotions	Morphed emotion identification
Verbal learning	Hopkins Verbal Learning Test–Revised	Verbal paired associates
Working memory	Letter–number sequencing	Backward digit span
Visual learning	Brief Visuospatial Memory Test–Revised	Hartshorne visual working memory
Reasoning and problem solving	NAB^d^: Mazes	Matrix Reasoning Test
Strategic risk taking^e^	N/A^f^	Balloon Analogue Risk Task
Attention	Continuous Performance Test–Identical Pairs	Multiple object tracking
Premorbid IQ^g^	National Adult Reading Test–Revised	Vocabulary

^a^Web-based tasks are shown in order of administration.

^b^BACS: Brief Assessment of Cognition in Schizophrenia.

^c^MSCEIT: Mayer–Salovey–Caruso Emotional Intelligence Test.

^d^NAB: Neuropsychological Assessment Battery.

^e^No equivalent offline measure was included.

^f^N/A: not applicable.

^g^No equivalent MCCB task; thus, the National Adult Reading Test was included for comparison.

#### Reference Battery

The MCCB was administered to participants as the reference battery in part 1 of the study to validate the web-based battery. The MCCB was created through the MATRICS initiative with the explicit aim of developing a consensus cognitive battery that could be used in schizophrenia research. The selection of tasks for the MCCB was driven by expert panels; consultations with scientists; evaluations of factor-analytic studies to identify relevant domains; and assessments of the psychometric properties, practicality, and tolerability of existing cognitive tasks [41,42]. The final MCCB comprises 10 tasks assessing 7 domains of cognition. In addition to the MCCB, the National Adult Reading Test (NART) was administered as a measure of premorbid IQ [21]. The NART is a measure of vocabulary that comprises 50 irregularly spelled words that the participant must read aloud, and it was included as a reference test for the web-based vocabulary test.

### Clinical and Demographic Variables

In addition to the cognitive assessment, participants answered questions about current diagnosis, medication, education, occupation, and current mood. The 12-item self-report version of the World Health Organization Disability Assessment Schedule Version 2 (WHODAS 2.0) was included as a measure of functioning [43]. The web-based questionnaire also included the Hospital Anxiety and Depression Scale [44] and the Altman Self-Rating Mania Scale [45]. Data on lifetime diagnosis, age of onset, and hospital admissions were obtained from the parent studies, CoMPaSS and NCMH (Multimedia Appendix 1).

### Part 1: Validation Study

#### Participants

Participants with major depressive disorder (15/65, 23%), bipolar disorder (type 1: 11/65, 17%; type 2: 5/65, 8%), or schizophrenia (15/65, 23%), as well as healthy controls (19/65, 29%) were recruited for the validation study. We selected these 3 diagnostic groups based on extensive research establishing the characteristics of cognitive performance of participants with each of these disorders using offline, traditional cognitive testing. For this study, we decided upon a conservative definition of depression that required a reported diagnosis and previous treatment with at least one antidepressant medication. This definition has been used in a study using self-reported measures of depression in the UKB [46]. Informed consent was obtained at both stages, in writing before administration of the MCCB and on the web before completing the web-based cognitive battery, and the participants were reimbursed for their participation.

#### Study Design

The participants were asked to complete two cognitive batteries on consecutive days: (1) the MCCB and the NART [21] and (2) the web-based battery (Table 1). A trained researcher administered the MCCB in a supervised setting on the first day, and then the participants were asked to complete the web-based battery unsupervised on the second day. The order of completion was not counterbalanced for practical reasons. First, some participants from the NCMH were recruited prospectively by completing the MCCB with a researcher immediately after completing the NCMH assessment; therefore, we could not randomly assign the order of completion. Second, it would have been difficult to ensure that the participants assigned to complete the web-based part first did so before their appointment as they were not supervised. The participants were asked to return a feedback questionnaire on completion of the study. They rated the overall web-based battery on enjoyment, duration, and difficulty and rated the clarity of the given instructions and information. They named the tasks that they liked the most and the least. They were also asked to provide information on any technical difficulties they experienced.

#### Data Analysis

All statistical analyses were conducted using R (version 3.3.0; R Foundation for Statistical Computing). Convergent validity was examined by conducting correlations between the MCCB and web-based tasks that assessed equivalent cognitive domains (Table 1). Correlations were conducted across the entire sample and for cases only. Pearson correlations were used when task performance was normally distributed, whereas Spearman correlations were used for tasks that were not normally distributed. A correlation matrix of all web-based tasks and the MCCB-equivalent tasks was also generated. Partial correlations were used to adjust for the time between completion of the batteries, age, and *g* (excluding domain of interest). Finally, correlation analyses were repeated after stratification by input device type (keyboard or touchscreen). Although previous studies do not appear to have corrected for multiple testing as the correlation coefficients are more important for validation [14-16], we calculated the corrected *P* values using the false discovery rate method.

### Part 2: Feasibility of Web-Based Cognitive Testing

#### Participants

Following completion of the validation study, participants from the NCMH and CoMPaSS were sent invitation letters or emails with instructions on how to participate in the study and their unique website link. A reminder was sent to participants who did not respond to our initial invitation. Response rates can be found in Multimedia Appendix 1. The collected data were combined with the validation data set. Participants were excluded from the analyses if they reported a neurological condition likely to affect cognitive function or if they did not complete any of the cognitive tasks. Diagnostic groups with >20 participants were included in the analyses. Healthy controls were excluded from the analyses if they reported a history of psychiatric diagnosis or medication or first-degree family history of schizophrenia, bipolar disorder, autism, or intellectual disability. Therefore, the final sample for analysis (N=887) included 21.1% (187/887) of controls, 16.5% (146/887) of participants with bipolar spectrum disorders, 4.8% (43/887) of participants with schizophrenia, 29.4% (261/887) of participants with unipolar depression, 7.6% (67/887) of participants with anxiety disorders, 5.4% (48/887) of participants with posttraumatic stress disorder, 2.4% (21/887) of participants with an eating disorder, and 12.9% (114/887) of participants who reported comorbid depression and anxiety disorders. The flow diagram in Multimedia Appendix 1 shows a breakdown of the number of participants excluded according to our criteria.

#### Data Analysis

##### Preparation of Cognitive Data

For each task, z scores were derived using the mean and SD of the controls (187/887, 21.1%). General cognitive performance *g* was derived using multidimensional scaling (MDS) for participants who had completed at least five tasks, as we have done in a previous study [47]. MDS is an analogous approach to principal component analysis; however, an advantage of this approach is that it can accommodate missing data [48,49]. General cognitive performance *g* was calculated as the first dimension produced by the MDS analysis. Measures of *g* derived from MDS and principal component analysis were highly correlated (*r*=0.996). All statistical analyses were conducted using R (version 3.3.0).

##### Sample Characteristics

We compared those who participated in part 2 of the web-based study (n=1152) with those who were invited but did not take part (nonresponders; n=5768) to assess whether there was recruitment bias in the web-based sample. These comparisons were conducted separately for cases and controls and were further separated by original study (NCMH or CoMPaSS). Logistic regressions were conducted with participation in the study as the outcome and the following predictors: age; sex; education; lifetime occupation; ethnicity; time since recruitment into parent study; and, among cases only, diagnosis, age of illness onset, and ever admitted to a psychiatric hospital.

##### Cognitive Performance and Functioning

We performed linear regressions with cognitive score as the predictor, total score on the WHODAS 2.0 as the outcome, and age and sex as covariates to test the association between cognitive performance and functioning. We repeated this regression by covarying for diagnosis. We ran separate linear regressions for each cognitive task and *g*. *P* values were corrected using the false discovery rate method.

##### Comparing Cognition Between Diagnostic Groups

We compared cognitive performance between healthy controls (187/887, 21.1%), major depressive disorder (295/887, 33.3%), bipolar disorder (116/887, 13.1%), and schizophrenia (38/887, 4.3%) using analysis of covariance with age and sex as covariates. Owing to the self-report nature of diagnoses in the NCMH, more conservative inclusion criteria were used to define depression, bipolar disorder, and schizophrenia in these analyses by considering medication use (see Multimedia Appendix 1 for details). Analyses of covariance were followed up with the honestly significant difference test by Tukey for pairwise comparisons. Hedge *g* effect sizes were calculated by dividing the mean group difference by the pooled SD [50].

### Statement of Ethical Approval

All participants in part 1 (validation study) provided written informed consent. All participants in part 2 (web-based only) were required to indicate their informed consent by selecting *yes* in response to the statement “I agree to take part in this study and know that I am free to leave the study at any point,” located on the information page of the study website. All stages of the web-based study titled *Cognition in Mood, Psychosis and Schizophrenia Study (CoMPaSS Web)* received ethical approval from the School of Medicine research ethics committee at Cardiff University (reference number: 15/64). The NCMH received a favorable ethical opinion from Wales research ethics committee 2 (reference: 16/WA/0323). CoMPaSS received a favorable ethical opinion from the South-East Wales research ethics committee panel (reference: 07/WSE03/110). All experiment protocols were conducted in accordance with the ethical standards of the Cardiff University School of Medicine research ethics committee and with the Declaration of Helsinki.

### Data Availability

The data sets generated and analyzed during this study are available at the Medical Research Council Centre for Neuropsychiatric Genetics and Genomics Walters Group Data Repository [51].

## Results

### Part 1: Validation Study

#### Data Completion and Sample Characteristics

Approximately 89% (58/65) of participants completed all the web-based tasks, and the same number completed all the MCCB tasks and NART (see Table 2 for individual domains). The sample had a wide age range (22 to 78, mean 47, SD 14.8 years) and a higher percentage of women (38/65, 58%) than men. More than one-third of the sample had an undergraduate degree (26/65, 40%; see Multimedia Appendix 1).

**Table 2:** Results of correlation analyses between web-based and offline tasks that assessed equivalent domains (N=65).

Domain	Participants, n (%)	Cases and controls			Cases only			Partial correlations^a^, *r*				Device, *r*	
		*r* (95% CI)	*P* value	*r* (95% CI)		*P* value	Time		Age	General cognitive performance (*g*)	Keyboard		Touchscreen
Speed of processing	65 (100)	0.73 (0.59 to 0.83)	<.001	0.69 (0.50 to 0.82)		<.001	0.74		0.66	0.39	0.75^b^		0.75^b^
Verbal learning^c^	63 (97)	0.41 (0.18 to 0.57)	.002	0.40 (0.11 to 0.64)		.02	0.42		0.36	0.19	0.48^b^		0.24
Working memory	64 (98)	0.34 (0.10 to 0.54)	.01	0.36 (0.07 to 0.59)		.03	0.33		0.31	0.1	0.3		0.43
Visual learning	63 (97)	0.12 (−0.13 to 0.36)	.35	0.15 (−0.15 to 0.43)		.33	0.12		0.02	−0.12	0.17		−0.01
Social cognition	63 (97)	0.26 (0.01 to 0.47)	.045	0.33 (0.04 to 0.56)		.04	0.26		0.24	0.1	0.17		0.48
Reasoning and problem solving^c^	64 (98)	0.53 (0.33 to 0.70)	<.001	0.55 (0.29 to 0.75)		<.001	0.53		0.41	0.26	0.55^b^		0.54
Attention	61 (94)	0.34 (0.09 to 0.55)	.01	0.31 (−0.01 to 0.56)		.06	0.34		0.27	0.08	0.36^b^		0.29
Premorbid IQ^c^	62 (95)	0.64 (0.44 to 0.78)	<.001	0.60 (0.38 to 0.76)		<.001	0.64		0.64	0.59	0.65^b^		0.66^b^
General cognitive performance (*g*)	65 (100)	0.78 (0.66 to 0.86)	<.001	0.79 (0.64 to 0.88)		<.001	0.78		0.72	N/A^d^	0.75^b^		0.85^b^

^a^Correlation coefficients after adjusting for time between completion of the 2 batteries in days, age, and *g* (cases and controls).

^b^Correlations significant after correction for multiple testing.

^c^Spearman rank correlation *ρ* shown instead because of nonnormal distribution for these tests.

^d^N/A: not applicable.

#### Convergent Validity

**Figure 1:**
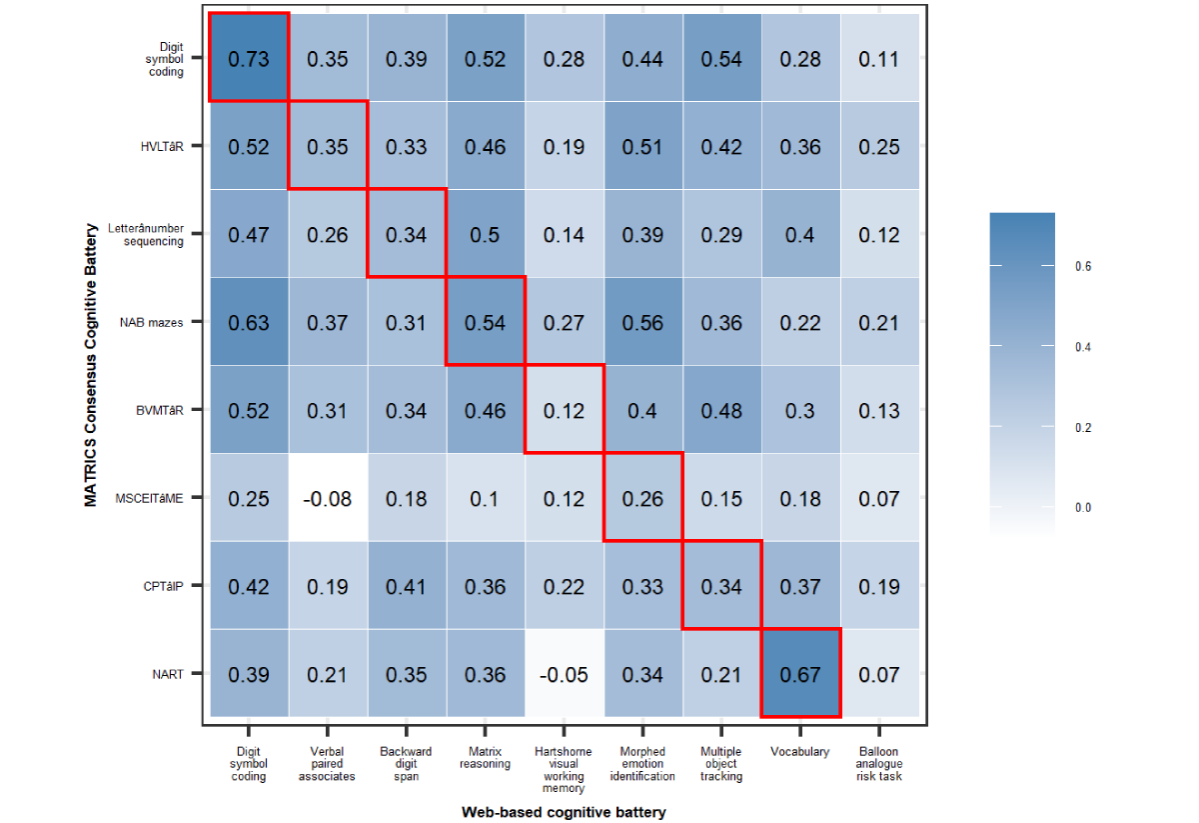
Pearson correlations between web-based and Measurement and Treatment Research to Improve Cognition in Schizophrenia Consensus Cognitive Battery (MCCB) tasks. Red squares indicate tasks assessing the same domain. Only tasks from the MCCB with an equivalent web-based task are shown (the Trail Making Test-A, Animal Naming Test, and Wechsler Memory Scale-III: Spatial Span were excluded). BVMT-R: Brief Visuospatial Memory Test–Revised; CPT-IP: Continuous Performance Test–Identical Pairs; HVLT-R: Hopkins Verbal Learning Test–Revised; MSCEIT-ME: Mayer–Salovey–Caruso Emotional Intelligence Test–Managing Emotions; NAB: Neuropsychological Assessment Battery; NART: National Adult Reading Test.

The results of correlations between tasks measuring equivalent domains are shown in Table 2 (performance on the MCCB and web-based battery can be found in Multimedia Appendix 1). The measures of *g* derived from the MCCB and web-based batteries were correlated (*r*=0.78). Scores from 88% (7/8) of the web-based tasks were correlated with scores from the MCCB equivalents (*r*=0.26-0.73). These results did not change when the time between completion of the batteries was added as a covariate, although correlation coefficients were attenuated after adjustment for age and *g*. When analyses were restricted to cases with a psychiatric diagnosis, scores from 75% (6/8) of the web-based tasks were correlated with scores from the MCCB equivalents (*r*=0.33-0.69). Similar correlations were observed when analyses were stratified by input device type (keyboard users: 47/65, 72%, *r*=0.17-0.75; touchscreen users: 17/65, 26%, *r*=−0.01 to 0.75); however, fewer correlations were significant because of reduced power. Scores on the Balloon Analogue Risk Task (BART) showed low correlations with the MCCB tasks (*r*=0.07-0.25; Figure 1).

#### Tolerability

Feedback questionnaires were received from 63% (41/65) of participants. Of those who responded, all participants agreed that the instructions given at the start of the study were clear and rated the clinical questionnaire positively. Overall, the cognitive tasks were rated as enjoyable by 100% (41/41) of the participants who responded and as being of reasonable duration and difficulty by 95%. Approximately 3% (2/65) of participants rated the duration and difficulty of the battery as *poor*. The most popular task was multiple object tracking, and the least popular was verbal paired associates. Of the 40 participants who responded to the question, 34 (85%) reported that they would be *more likely* to take part in future web-based studies after taking part in this study, whereas 5 (13%) responded *don’t know*, and 1 (3%) responded that they were *less likely*. Of the 40 participants, 6 (15%) reported technical difficulties and, of these 6 participants, 5 (83%) were able to complete all the tasks; thus, this did not affect the availability of data for these participants.

### Part 2: Feasibility of Web-Based Cognitive Testing

#### Sample Characteristics

We compared the demographic and clinical characteristics of all participants recruited during part 2 of the study (n=1152) and nonresponders (n=5768; see Table 3 for cases and Table 4 for controls) to evaluate whether recruitment bias was present in the web-based sample. In CoMPaSS, web-based participants were more highly educated than nonresponders (odds ratio [OR] 1.49, 95% CI 1.15-1.98; *P*=.004). Among NCMH cases, the significant predictors for web-based participation were diagnosis of eating disorder (OR 2.17, 95% CI 1.17-4; *P*=.01), diagnosis of comorbid depression and anxiety (OR 5.12, 95% CI 3.38-7.83; *P*<.001), absence of diagnosis of schizophrenia (OR 0.59, 95% CI 0.35-0.94; *P*=.03), older age (OR 1.01, 95% CI 1-1.02; *P*=.003), being female (OR 1.3, 95% CI 1.07-1.58; *P*=.009), ethnicity other than White European (OR 0.66, 95% CI 0.46-0.96; *P*=.03), younger age of onset (OR 0.99, 95% CI 0.98-0.99; *P*=.001), higher level of education (OR 1.19, 95% CI 1.11-1.29; *P*<.001), and shorter time since recruitment into the NCMH (OR 0.98, 95% CI 0.97-0.98; *P*<.001). Among the controls, older age (OR 1.03, 95% CI 1.01-1.04; *P*<.001), higher levels of education (OR 1.5, 95% CI 1.19-1.83; *P*<.001), and shorter time since recruitment into the NCMH (OR 0.95, 95% CI 0.93-0.96; *P*<.001) were associated with web-based participation.

**Table 3:** Characteristics of participants and nonresponders (cases; N=5981)^a^.

Characteristic		NCMH^b^ cases						CoMPaSS^c^ cases							
		Took part (n=906)	Did not take part (n=4341)	OR^d^ (95% CI)		*P* value		Took part (n=33)		Did not take part (n=701)		OR^d^ (95% CI)		*P* value	
Age (years), mean (SD)		47.82 (14.6)	47.45 (14.77)	1.01 (1-1.02)		.003		50.33 (12.63)		52.19 (12.42)		0.99 (0.96-1.03)		.71	
Women, n (%)		668 (73.7)	2838 (65.4)	1.3 (1.07-1.58)		.009		18 (54.5)		283 (40.4)		1.47 (0.68-3.17)		.32	
**Ethnicity, n (%)**					0.66 (0.46-0.96)		.03						0.25 (0.05-1.81)		.11
	White European	853 (94.2)	4105 (94.6)					>28 (>84.8)		670 (95.6)					
	Other ethnicities	53 (5.8)	236 (5.4)					<5 (<10)		14 (2)					
**Highest qualification, n (%)**					1.19 (1.11-1.29)		<.001						1.49 (1.15-1.98)		.004
	None	29 (3.2)	301 (6.9)					<5 (<10)		164 (23.4)					
	Any qualifications	861 (95)	2918 (67.2)					>28 (>84.8)		506 (72.2)					
	Degree	412 (45.5)	1098 (25.3)					8 (24.2)		114 (16.3)					
**Lifetime occupation, n (%)**					1.04 (0.86-1.27)		.69						0.57 (0.2-1.47)		.26
	Professional	344 (38)	985 (22.7)					5 (15.2)		70 (10)					
	Other occupations	527 (58.2)	2167 (49.9)					28 (84.8)		559 (79.7)					
	Never worked	29 (3.2)	84 (1.9)					0 (0)		53 (7.6)					
≥1 admission, n (%)		238 (26.2)	1298 (29.9)	1.05 (0.85-1.3)		.66		28 (84.8)		624 (89)		0.86 (0.27-3.88)		.82	
Age of onset (years), median (IQR)^e^		17 (15)	19 (16)	0.99 (0.98-0.99)		.001		19 (13.5)		20 (11)		1 (0.95-1.05)		.99	
Months since recruitment into parent study, median (IQR)^e^		38 (33)	48 (39)	0.98 (0.97-0.98)		<.001		108.5 (23.25)		110 (23.75)		1 (0.98-1.02)		.95	

^a^Not all cells add up to the total N because of missing data.

^b^NCMH: National Centre for Mental Health.

^c^CoMPaSS: Cognition in Mood, Psychosis, and Schizophrenia Study.

^d^OR: odds ratio.

^e^Median and IQR are shown because of nonnormal distribution.

**Table 4:** Characteristics of participants and nonresponders (controls; N=939)^a^.

Characteristic		NCMH^b^ controls					
		Took part (n=213)	Did not take part (n=726)	OR^c^ (95% CI)		*P* value	
Age (years), mean (SD)		54.58 (17.24)	48.65 (17.69)	1.03 (1.01-1.04)		<.001	
Women, n (%)		139 (65.3)	507 (69.8)	0.99 (0.65-1.52)		.97	
**Ethnicity, n (%)**					1.19 (0.46-3.37)		.72
	White European	205 (96.2)	673 (92.7)				
	Other ethnicities	8 (3.8)	53 (7.3)				
**Highest qualification, n (%)**					1.5 (1.19-1.83)		<.001
	None	<5 (<2.3)	7 (1)				
	Any qualifications	>200 (>93.9)	302 (41.6)				
	Degree	134 (62.9)	163 (22.5)				
**Lifetime occupation, n (%)**					0.71 (0.44-1.13)		.15
	Professional	103 (48.4)	142 (19.6)				
	Other occupations	99 (46.5)	167 (23)				
	Never worked	6 (2.8)	<5 (<1)				
Months since recruitment into parent study, median (IQR)^d^		25 (10)	28 (13)	0.95 (0.93-0.96)		<.001	

^a^Not all cells add up to the total N because of missing data.

^b^NCMH: National Centre for Mental Health.

^c^OR: odds ratio.

^d^Median and IQR are shown because of nonnormal distribution.

#### Completion Rates

Of the 961 participants who met the inclusion criteria, 887 (92.3%) completed at least one web-based cognitive task, and 644 (67%) completed all the tasks in the web-based battery. A breakdown of completion rates for each task and by diagnostic group can be found in Multimedia Appendix 1.

#### Cognitive Performance and Functioning

Linear regression indicated that lower cognitive performance (*g*) was associated with higher WHODAS 2.0 scores, indicating poorer functioning (B=−1.76, SE 0.26; *P*<.001). This association remained significant after covarying for diagnosis (B=−1.64, SE 0.26; *P*<.001). Higher scores on all tasks were associated with better functioning (Table 5).

**Table 5:** Association between cognitive performance and functioning (N=961).

Variable			Participants, n (%)		B^a^ (SE)		*P* value
General cognitive performance (*g*)			561 (58.4)		−1.76 (0.26)		<.001
General cognitive performance (*g*) after covarying for diagnosis			561 (58.4)		−1.64 (0.26)		<.001
**Individual tasks**							
	Digit symbol coding	645 (67.1)		−3.08 (0.47)		<.001	
	Verbal paired associates	557 (58)		−1.72 (0.50)		<.001	
	Backward digit span	528 (54.9)		−2.03 (0.51)		<.001	
	Hartshorne visual working memory test	499 (51.9)		−1.73 (0.51)		.008	
	Morphed emotion identification	565 (58.8)		−1.78 (0.53)		<.001	
	Matrix Reasoning Test	489 (50.9)		−1.54 (0.43)		<.001	
	Balloon Analogue Risk Task	487 (50.7)		−1.29 (0.39)		<.001	
	Multiple object tracking	459 (47.8)		−1.92 (0.43)		<.001	
	Vocabulary	470 (48.9)		−1.51 (0.51)		.003	

^a^Regression coefficient.

#### Comparing Cognition Between Diagnostic Groups

There was a significant main effect of diagnosis for *g* (*F*_3,511_=21.89; *P*<.001). Participants with depression had a lower performance than the controls (Hedges *g*=0.32; *P*=.01). The bipolar disorder group had lower performance than the controls (Hedges *g*=0.65; *P*<.001) and the depression group (Hedges *g*=0.33; *P*=.03). Participants with schizophrenia had the lowest performance relative to the controls (Hedges *g*=1.36; *P*<.001) and had lower performance than participants with depression (Hedges *g*=1.04; *P*<.001) and bipolar disorder (Hedges *g*=0.71; *P*=.002). Domain-specific effect sizes for pairwise comparisons are shown in Multimedia Appendix 1.

## Discussion

### Principal Findings

We developed a web-based cognitive battery for use in psychiatric research. The battery was designed to test the domains specified by the National Institute of Mental Health’s MATRICS initiative. The aims of this study were to validate the web-based battery against the MCCB and evaluate whether web-based cognitive testing is suitable for research on psychiatric disorders. Our principal findings for each part of the study are outlined in the sections below.

### Validation of the Web-Based Battery

We assessed convergent validity by conducting correlations between tasks that measured equivalent domains. Approximately 88% (7/8) of web-based tasks were correlated with the MCCB equivalent (*r*=0.26-0.73). This is comparable with the correlation coefficients reported in the validation of the UKB tasks (*r*=0.22-0.83) [16], web-based Cambridge Neuropsychological Test Automated Battery (*r*=0.39-0.73) [14], Amsterdam Cognition Scan (*r*=0.36-0.78) [15], and NutriCog Battery (*r*=0.42-0.73) [52]. However, only digit symbol coding, matrix reasoning, and vocabulary were most highly correlated with their equivalent offline tasks, and even these tasks were correlated with other MCCB tasks. The correlations between equivalent domains were also attenuated and, in some domains, close to 0 after correction for *g*. These findings suggest that at least some of the correlations between the web-based tasks and the MCCB were nonspecific and may reflect the tendency of cognitive tasks to be at least moderately correlated with a large proportion of the variance in performance accounted for by a higher-order factor (*g*) [53]. Overall, the battery may be better suited as a measure of general cognitive function *g* rather than a measure of specific cognitive domains.

There are two key differences between the web-based battery and the MCCB: (1) website versus offline administration and (2) use of different tasks to measure the same domain. Both differences are likely to affect the magnitude of the correlations between the tasks. Examining the correlation for speed of processing provides some insight into the extent to which the correlations are affected by these differences as the speed of processing domain was measured using offline and web-based versions of the same task, digit symbol coding. These tasks were the most highly correlated (*r*=0.73), suggesting that differences in web-based and traditional administration may not have had such a large impact on the magnitude of the correlations as differences arising from different tasks being used. This is consistent with the findings from the UKB and the Amsterdam Cognition Scan, showing that tasks with differing methodologies had lower correlations than those with similar methodologies [15,16]. A further consideration is the delay between administering the first and second batteries, as some participants did not complete the web-based battery on the second day as instructed. The correlations between the MCCB and the web-based battery did not change after controlling for the delay between the completion of the batteries, suggesting that the delay had little influence on the relationship between the tasks.

We observed the lowest nonspecific correlations between tasks that used different methodologies. For example, the morphed emotion identification task had a low correlation with the measure of social cognition in the MCCB, the Mayer–Salovey–Caruso Emotional Intelligence Test–Managing Emotions (MSCEIT-ME; *r*=0.26), but higher correlations with all other MCCB measures (*r*=0.33-0.56). Morphed emotion identification and MSCEIT-ME are designed to tap different aspects of social cognition. Morphed emotion identification is designed to measure a participant’s ability to recognize emotional facial expressions [22], whereas the MSCEIT-ME was developed to measure emotion self-regulation [54]. We did not select a formally equivalent web-based task for the MSCEIT-ME for 2 reasons. First, although previous research has identified impaired performance on the MSCEIT-ME in participants with schizophrenia [19,55], studies have not identified impairments in this task for participants with bipolar disorder [19,56,57]. Therefore, we did not consider it a suitable measure for this web-based battery, which was designed to measure cognitive function in participants with a range of psychiatric disorders. Second, in our experience of administering the MSCEIT-ME to >1000 participants with psychosis, we found that participants frequently required guidance and explanations of the scenarios, which would not be practical for a web-based, unsupervised measure. Studies have identified impairments in emotion recognition in participants with bipolar disorder [58,59], depression [60], autism [61], and posttraumatic stress disorder [62]. Therefore, we considered this a more suitable measure for cross-disorder research. However, we did not find evidence of impairments in this task for participants with depression or bipolar disorder in this study as effect sizes were small (*d*=0.17 for depression; *d*=0.28 for bipolar disorder) and nonsignificant (see Multimedia Appendix 1).

The Hartshorne visual working memory task was not correlated with its selected equivalent in the MCCB, the Brief Visuospatial Memory Test–Revised (BVMT-R). The BVMT-R is an immediate visual recall task in which participants are presented with shapes for 10 seconds and asked to draw these shapes from memory [63]. Drawings are rated based on the accuracy of recall and memory of the location of the shapes. It was not possible to select a task that would entirely replicate the BVMT-R because of the difficulties in automating the study administration and scoring on the web and the possibility that participants may cheat in an unsupervised setting by copying the shapes while they are being displayed onscreen. Therefore, the Hartshorne visual working memory task was chosen as an alternative as performance on this task also relies on short-term memory of both shapes and locations. However, the Hartshorne visual working memory task is more complex as, in addition to memorizing shapes and locations, the participant must identify whether a new target shape is the same or different from the shape that previously occupied that position [23]. As such, the task incorporates aspects of working memory and problem solving. Our results suggest that these tasks are not comparable and that the Hartshorne visual working memory task is not a suitable alternative measure of immediate visual recall.

The BART was not correlated with any of the MCCB tasks. This was not surprising, given that the BART is a behavioral measure rather than a neurocognitive task, and the MCCB is primarily made up of neurocognitive measures. Nevertheless, the BART may be a useful measure of risk-taking behavior that does not rely on self-report, as it has been shown to be correlated with measures of sensation seeking, impulsivity, behavior constraint, and actual risk-taking behaviors [24].

### Feasibility of Web-Based Cognitive Testing

We demonstrated that web-based cognitive testing is an effective method of collecting data from a large sample of participants with psychiatric disorders. To date, we have obtained cognitive data from >1000 participants diagnosed with a range of psychiatric disorders. A key concern with web-based testing in psychiatric research is whether the sample is representative, particularly given evidence that there is a digital divide between patients with psychiatric disorders and the wider UK population [64]. Across all samples (CoMPaSS and NCMH cases and controls), a higher level of education was associated with web-based participation. This finding suggests that earlier concerns about education bias in web-based samples may be correct and is a clear limitation of web-based testing. This issue is not exclusive to web-based cognitive testing, as similar recruitment biases have been identified in mental health studies using web-based questionnaires [17], and response rates to research invitations have been shown to be positively associated with educational attainment more generally [65]. The web-based control group was older and had been recruited more recently into the NCMH than nonresponders. Finally, participants with psychiatric disorders recruited from the NCMH were older, more likely to be female, less likely to be White European, and had been recruited more recently than nonresponders. The OR for age was close to 1, which does not support early preconceptions that internet samples would be overrepresented by younger people [11]. Our web-based sample was drawn from 2 existing clinical studies, and it should be noted that these original studies also have recruitment bias, although there is some evidence that the CoMPaSS sample is representative of the wider population of patients with psychosis in Wales based on the linkage of these data with routinely collected records [66]. Nevertheless, we were unable to assess whether the participants in our web-based study were representative of the wider population of patients with psychiatric disorders. However, our results do indicate that web-based samples may have recruitment bias beyond that seen in traditional clinical studies of psychiatric disorders.

In terms of clinical differences between participants and nonresponders, we did not find evidence of differences in hospital admissions. The difference in age of onset was significant; however, the OR was close to 1, indicating that the difference was very small. There were differences in the proportion of diagnoses, as individuals with schizophrenia were less likely to participate, and individuals with an eating disorder or comorbid depression and anxiety were more likely to participate. The response rates for individuals with other psychotic disorders were also low. These response rates may reflect the severity of illness. We did not find differences in hospital admissions as a binary measure but were unable to examine the number of admissions, length of hospitalizations, or whether admissions were under the Mental Health Act (as data were only available for a small proportion of the sample), which would have provided more detailed information on the severity of illness. Although this is another limitation of web-based testing in a mental health sample, an advantage of web-based testing is that the tests can be administered anywhere with internet access, supervised or unsupervised. Sample representativeness may be improved by providing opportunities for participants to take part in a supervised setting, such as a psychiatric clinic or research facility, if they lack the skills or resources to access the internet unsupervised. A combination of approaches (supervised and unsupervised and clinical or home settings) may reduce the financial and logistical burden of assessing cognitive function in large cohorts associated with traditional studies while limiting the recruitment bias associated with purely web-based studies. Studies using web-based methods for data collection should consider providing additional support to individuals with more severe mental illnesses, such as psychosis.

The completion rates give an indication of the tolerability of the cognitive battery. Of the 961 eligible participants who consented to the study, 887 (92.3%) completed at least one task, which is similar to the 87% reported by another web-based cognitive study [67]. Most participants completed all 9 tasks in the cognitive battery (644/961, 67% of eligible participants or 644/887, 72.6% of participants who started the tasks). This figure is comparable with the completion rates reported by the Twins Early Development Study for their web-based battery of 8 tasks (65%), although the study assessed children [9]. This figure is lower than those reported in face-to-face cognitive studies of participants with psychiatric disorders [68,69]. A lower completion rate was expected, given that the participants were unsupervised and would not have the support of a researcher to complete the study. However, this should be considered a potential limitation of web-based testing in psychiatric research, and more work is needed to understand who is likely to drop out; the reasons for dropout; and whether any measures can be taken to mitigate dropout; for example, by reducing the overall length of the battery. Overall, the completion rates were adequate, suggesting that the tasks were well-tolerated by most participants. All participants who did not complete the tasks were followed up by email or phone, and technical issues were recorded and resolved where possible. The number of technical issues reported by participants was small (part 1: 6 issues reported; part 2: 9 issues reported). In total, 19 participants reported technical problems, of which 13 (68%) were able to complete the battery (part 1: 6, 32% reported [9% of validation sample] and 5, 26% completed; part 2: 13, 68% reported [1.5% of sample] and 8, 42% completed).

Lower cognitive performance was associated with poorer functioning. These results suggest that performance on the battery is an important indicator of overall functioning. The results are consistent with a prospective study that reported an association between cognitive performance and WHODAS 2.0 scores in a cross-disorder sample of participants with depression, bipolar disorder, and psychosis [70]. In this study, performance on digit symbol coding and backward digit span was most strongly associated with scores on the WHODAS 2.0. Both digit symbol coding and backward digit span have short administration times and thus may be particularly suited for brief assessments of cognition in a clinical setting. It should be noted that this study was cross-sectional; thus, it was not possible to examine whether changes in performance on the battery were associated with changes in functioning.

We compared cognitive performance in participants with major depressive disorder, bipolar disorder, and schizophrenia as these groups have been extensively assessed for research using traditional cognitive assessments. Our results demonstrated a pattern of decreasing cognitive scores from major depressive disorder to bipolar disorder to schizophrenia and are consistent with studies showing lower performance in schizophrenia compared with major depressive disorder and bipolar disorder [70,71]. This suggests that the performance of the battery can be used to discriminate cases and controls. However, this pattern was not consistent in the analyses of individual domains. Effect sizes for the depression group across domains (Hedges *g*=0.07-0.39) were also lower than those reported by previous meta-analyses (effect sizes ranged from 0.32 to 0.97 across domains [3,72,73]), which may be explained by recruitment bias or the self-report nature of the NCMH diagnoses.

### Limitations

In addition to the limitations discussed above, several further limitations should be noted. Test–retest reliability of the web-based battery was not assessed in this study. In assessing validity, the correlation between a new task and the gold standard task cannot exceed √(reliability of reference task×reliability of new task) [10]. Therefore, the upper limits of the correlations between tasks were unknown. This is helpful for interpretation but does not change the magnitude or significance of the correlations. The order of completion of the batteries was not counterbalanced in the validation study for practical reasons, which may mean that performance on the web-based battery was subject to practice effects, particularly those tasks with similar methodologies. However, we would expect practice effects to be minimal as most web-based and MCCB tasks used different methodologies, and none of the tasks used the same stimuli. In the NCMH sample, diagnosis was based on self-report rather than structured interviews, which may result in incomplete or inaccurate diagnoses. However, participants were asked to report diagnoses that they had been given by a health professional, which is consistent with the approach taken by other large studies with self-report measures of diagnosis, such as the UKB [25,26]. There was a smaller schizophrenia group because of a lower response rate from participants with this disorder. A further limitation of web-based testing is the unsupervised environment in which the data are collected, which makes it difficult to minimize distractions or cheating. A study by Germine et al [12] using the same platform (TestMyBrain) found low levels of self-reported cheating, and their data were not consistent with widespread cheating. We selected tasks that would minimize the possibility of cheating where possible and also instructed participants to complete the tasks in a quiet environment. In addition, most participants completed both batteries at home to minimize the differences in the test settings.

### Conclusions

The web-based battery has several strengths, including the use of tasks taken from published research, domains selected based on the MATRICS initiative, and compatibility with a range of devices, including touchscreen devices. The availability of demographic and clinical data on individuals who did not participate in the study was a unique strength that allowed us to assess potential recruitment bias.

In conclusion, we developed a new web-based cognitive battery and used it to collect data from a large sample of participants with a range of psychiatric disorders. There was some evidence of recruitment bias, and the levels of impairment found in the depression group were less severe than those reported by traditional face-to-face studies. However, the battery provided a reliable measure of *g*, completion rates were adequate, our findings were consistent with studies using traditional assessments, and feedback from participants was positive. Our findings offer valuable insights into the advantages and disadvantages of testing cognitive function remotely for mental health research, which is particularly important given the increasing number of psychiatric studies using digital methods of assessment. In the next stage of development, we intend to use these findings to reduce the size of the battery to a briefer version removing tasks with low correlations, rated poorly by participants, or those presenting technical issues. Given that the correlations between some web-based tasks and the MCCB were nonspecific, the battery will include the tasks that were best suited for measuring their domain and providing a measure of *g*. We will also redesign the battery with a user-friendly interface with input from patient representative groups.

Cognitive impairments are one of the causes of long-term disability among patients with psychiatric disorders, particularly schizophrenia [7,8]. In the United Kingdom, assessment of cognitive skills has been recommended in clinical settings for patients with psychosis [74]. However, there are barriers associated with accessing appropriate cognitive assessments, such as cost, licensing, and the lack of available assessments that provide clinically meaningful feedback on performance [75]. It has been suggested that web-based tools may be a cost-effective solution [75]. Our results indicate that this assessment is suitable for detecting impairments in individuals with schizophrenia. Therefore, it is our intention to explore the potential clinical utility of the battery in future work.

## Appendix

Multimedia Appendix 1 Detailed information about the parent studies, recruitment response rates, inclusion criteria, tasks included in the web-based battery, completion rates, and supplementary results.

## Abbreviations

BART: Balloon Analogue Risk Task
BVMT-R: Brief Visuospatial Memory Test–Revised
CoMPaSS: Cognition in Mood, Psychosis, and Schizophrenia Study
MATRICS: Measurement and Treatment Research to Improve Cognition in Schizophrenia
MCCB: Measurement and Treatment Research to Improve Cognition in Schizophrenia Consensus Cognitive Battery
MDS: multidimensional scaling
MRC: Medical Research Council
MSCEIT-ME: Mayer–Salovey–Caruso Emotional Intelligence Test–Managing Emotions
NART: National Adult Reading Test
NCMH: National Centre for Mental Health
OR: odds ratio
UKB: UK Biobank
WHODAS 2.0: World Health Organization Disability Assessment Schedule Version 2
