# Supplementary material for: Web-Based Cognitive Testing in Psychiatric Research: Validation and Usability Study
Source: J Med Internet Res. 2022 Feb 10;24(2):e28233. doi: 10.2196/28233 (PMC8874806; doi:10.2196/28233)
Supplement: Multimedia Appendix 1 [file jmir_v24i2e28233_app1.docx]

**Title**:

Web-based Cognitive Testing in Psychiatric Research: Validation and Usability Study

**Running Title**: Web-based Cognitive Testing in Psychiatric Research

**Authors**:

Amy J Lynham, PhD, Ian R Jones, MRCPsych, PhD, James TR Walters, MRCPsych, PhD

**Affiliations**:

MRC Centre for Neuropsychiatric Genetics and Genomics, Division of Psychological Medicine and Clinical Neurosciences, School of Medicine, Cardiff University, Cardiff, United Kingdom

**Corresponding Author**:

James T.R. Walters

MRC Centre for Neuropsychiatric Genetics and Genomics

Division of Psychological Medicine and Clinical Neurosciences, Cardiff University School of Medicine

Hadyn Ellis Building

Maindy Road

Cardiff

CF24 4HQ

Email: WaltersJT@cardiff.ac.uk

# Supplementary Methods

## Methods S1: Original Cohorts

### National Centre for Mental Health (NCMH)

The National Centre for Mental Health (NCMH) is a Welsh Government-funded Research Centre that investigates neurodevelopmental, adult, and neurodegenerative psychiatric disorders across the lifespan. The centre is operated by Cardiff, Swansea and Bangor Universities, in partnership with NHS Health Boards across Wales. The aim is to facilitate a Wales-wide collection of biological samples, clinical data, and demographic information, with the long-term vision of improving the diagnosis and management of mental illness. The cohort of participant volunteers includes individuals over the age of 4 years, who have experienced, or are related to someone who has experienced, a childhood developmental disorder, a major mood and/or psychotic disorder, post-traumatic stress disorder, a learning disability, a personality disorder and/or a neurodegenerative disorder. To allow comparisons, the cohort includes control participants who have not experienced any of the disorders above, and may or may not be related to someone who has.

Participants were recruited using a variety of systematic approaches in primary, secondary and tertiary health care services, including (a) the identification of potential participants by clinical care teams, (b) screening of clinical notes, and (c) the use of disease registers. Non-systematic recruitment approaches included advertising in local media, placing posters and leaflets in NHS waiting areas, liaising with voluntary organisations and contacting individuals enrolled in previous studies within the Institute of Psychological Medicine and Clinical Neurosciences. Potential participants were provided with a copy of the patient information sheet before being asked to consent to take part. All adult participants with sufficient mental capacity provided informed consent to participate. Children under the age of 16 and adults with insufficient mental capacity provided assent (where possible) and consent was obtained from a nominated consultee such as the next of kin, a family member, or a carer. Participants were administered a brief questionnaire either online or via interview with a trained researcher to ascertain details related to the participant’s personal and family history of mental illness and their current medication profile. Participants are asked the question, “Has a doctor or health professional ever told you that you have any of the following diagnoses?” Participants are given a list of diagnoses and asked to indicate all diagnoses that apply. They are then asked to indicate which of the diagnoses they have ticked would they consider to be their primary, secondary and tertiary diagnoses. Finally, they are asked the question, “If we were to speak to your clinical team or general practitioner, would they agree with that?” This is consistent with the approach taken by other large studies with self-report measures of diagnosis, such as the UK Biobank [25, 26]. A sample of venous blood (15-50ml for adults, 10-30 for children) was taken for genetic and other analyses. Where this was not possible, a sample of saliva was obtained. Participants were given a pack of standardized self-report questionnaires to complete and return to the research team by post after the initial assessment. Information regarding diagnosis, symptoms, treatment and outcomes was obtained from clinical records where appropriate consent had been obtained to do so. Participants were able to consent to being contacted regarding future research opportunities*.*

A favourable ethical opinion was received from Wales Research Ethics Committee 2 on 25^th^ November 2016.

### Cognition in Mood, Psychosis and Schizophrenia Study (CoMPaSS)

CoMPaSS is a locally recruited sample of individuals with schizophrenia and related psychosis from South Wales in the UK [19]. This sample includes participants previously referred to as the Cardiff Cognition in Schizophrenia sample (CardiffCOGS, described elsewhere by Rees and colleagues [27]). Participants were interviewed using the Schedule for Clinical Assessment in Neuropsychiatry [20]. This interview was reviewed along with available clinical records by trained raters to determine consensus lifetime DSM-IV and ICD-10 diagnoses. All participants provided written informed consent and were reimbursed for their participation. Participants were assessed for capacity to provide informed consent by their clinical team and an appropriately trained researcher. The study had UK multi-site NHS ethical approval.

## Methods S2: Full Details of Online Tasks

All tasks were selected from and hosted on The Many Brains Project’s online cognitive testing platform, “testmybrain.org” [12, 28]. Tasks were selected to measure, as closely as possible, the domains assessed by the MATRICS Consensus Cognitive Battery.

### Speed of processing: Digit Symbol Coding

This task is an adapted version of the well-validated measure of processing speed [29]. Full details of the online version can be found in Passell et al. [28]. During the task, a key of nine symbols and corresponding numbers is shown at the top of the screen. Participants must select the number that corresponds to each target symbol (“1”, “2” or “3”). The outcome measure was the number of correct responses in 90 seconds. This task was selected as our measure of processing speed, as Digit Symbol Coding (BACS version) is included in the MCCB.

### Social cognition: Morphed Emotion Identification

The measure of social cognition included in the MCCB is the Mayer-Salovey-Caruso Emotional Intelligence Test – Managing Emotions subtest (MSCEIT-ME), a measure of emotion regulation and intelligence [36]. A similar task was not available on the TestMyBrain platform. In addition, our experience of delivering the MSCEIT-ME in our previous research was that participants often required guidance to interpret the scenarios, which would not be possible online. From the social cognition tasks available on the TestMyBrain platform, we selected the Morphed Emotion Identification task, as impairments in emotion recognition have been demonstrated across a range of psychiatric disorders.

This task uses the face datasets developed by Perrett and colleagues at the University of St. Andrews, United Kingdom [30, 22]. Participants are presented with a face and must decide whether the face looks angry, fearful, happy or disgusted. Faces are morphed between a neutral face and each emotion at varying intensities. The outcome measure was the number of correct responses out of 60 faces.

### Verbal learning: Verbal Paired Associates

There were specific challenges with selecting a suitable measure of verbal learning that would be practical in an unsupervised, online setting. The MCCB includes the Hopkins Verbal Learning Test – Revised (HVLT-R) as a measure of verbal learning. The HVLT-R is a free immediate recall task of a spoken list of twelve words. To conduct this task in an online setting, the participants would need access to speakers and a microphone, which increases the possibility of technical difficulties and may limit participation based on the technology the participant has available to them. Alternatively, the participants could have typed their responses but this could negatively impact participants who struggle with spelling or typing. In both cases, the responses would need to be scored manually rather than automatically, thereby limiting the batteries use for collection of large samples. Measures of paired associate learning have been shown to load onto the same factor as list learning tasks in factor analytic studies [31]. Therefore, the Verbal Paired Associates task was included as an alternative.

A set of 25 unrelated word pairs are presented on the screen in the learning phase. During the test phase, participants are presented with the first word from each previously presented word pair and must select the second word from four options. The outcome measure was the number of correct responses out of 25 word pairs. Full details of the task can be found in Passell et al. [28].

### Working memory: Backward Digit Span

Initially an online version of the Letter-Number Sequencing task from the MCCB was selected as a measure of working memory. However, due to technical problems in developing a mobile version of the task, the task was replaced with the Backward Digit Span. Scores on Backward Digit Span have been shown to load onto the same factor as Letter-Number Sequencing in factor analytic studies [31].

The Backward Digit Span task is a frequently used measure of working memory [32]. Participants are presented with a sequence of numbers and must recall them in the reverse order. The lengths of the sequences increase every two trials until the participant is no longer able to correctly recall the sequence backwards. The outcome measure was the maximum length of number sequence the participant was able to recall backwards.

### Visual learning: Hartshorne Visual Working Memory

The MCCB includes the Brief Visuospatial Memory Test – Revised (BVMT-R) as a measure of visual learning. Like the HVLT-R, the BVMT-R is an immediate free recall task, which would be difficult to replicate online. Therefore, a measure of visual recognition was selected as an alternative, the Hartshorne Visual Working Memory task. Although the methodologies of the two tasks differ substantially, there are some similarities. Performance on both tasks rely on memory for both shape type and location.

The Hartshorne Visual Working Memory task was selected as a measure of visual learning [23]. In this task, four shapes are presented at four positions around a central cross. These objects are then replaced with one object in the location of one of the previous objects. The participant must decide whether that object is the same or different than the object that was previously in that location. The outcome measure was the number of correct responses out of 42 trials.

### Reasoning and problem solving: Matrix Reasoning Test

The MCCB reasoning and problem-solving task, NAB Mazes, was not available on the TestMyBrain platform and would have been difficult to replicate online. The Matrix Reasoning test was selected as an alternative as previous factor analytic studies have shown that this test loads highly onto a reasoning and problem solving or executive function dimension [31].

This task is based on the well-validated Matrix Reasoning test used in the Wechsler Abbreviated Scale of Intelligence II [33]. Full details of the online task can be found in Passell et al. [28].A set of images is shown on the screen that follows a logical rule. The participant must determine the rule and select the image that best completes the set. The outcome measure was the number of correct responses out of 35 trials.

### Strategic Risk Taking: Balloon Analogue Risk Task

Over 30 trials, the participant is presented with a series of balloons that have different popping thresholds [24, 34]. For each trial, the participant must blow up the balloon by clicking on a button and decide when to stop and cash in their points. The larger the balloon at the end of the trial, the more points a participant is rewarded but no points are given if the balloon pops. The primary outcome measure of the BART is the number of points awarded although the number of times the balloon pops is also recorded. The outcome measure was the number of points collected by inflating 30 balloons. Higher scores indicate more effective strategic risk-taking.

### Attention: Multiple Object Tracking

The Multiple Object Tracking paradigm was developed as a measure of visual cognition [28, 35]. In this task, participants must follow multiple targets as they move across the screen amongst other identical objects. This task has several advantages over other sustained attention tasks such as the Continuous Performance Test, which is included in the MCCB. The task involves attending to multiple moving objects rather than a single object and this is thought to be more characteristic of real-world attention. The task requires active attention to target objects rather than passive vigilance. This may be beneficial for keeping participants engaged in the task in an unsupervised setting. The outcome measure was the number of correct responses over 30 trials. Within a trial, each correctly identified target is one point.

### Premorbid IQ: Vocabulary

A measure of premorbid IQ was included in addition to the domains measured by the MCCB. Vocabulary is thought to be preserved after onset of a psychiatric disorder and is used as an estimate of premorbid IQ [36, 37]. In this task, participants are shown a target word and asked to select which of four words is closest in meaning to the target word [28, 38, 39]. There are twenty words in total and the total score was used as our outcome measure for this task.

## Methods S3: Additional data from parent studies

Data on lifetime diagnosis, age of onset and history of hospital admissions were obtained from the parent studies, CoMPaSS and NCMH. In CoMPaSS, age of onset was defined as the age at which the participant’s symptoms caused impairment in their life. Impairment was defined as: 1) disruption in work or social life more or less completely, 2) fights or other violence, 3) job loss or unable to work, 4) police involvement, 5) family separation, 6) hospital admission or 7) receiving specific treatment. This was rated according to participants’ responses to the SCAN interview and review of clinical records. In NCMH, participants were asked to report the age at which they first developed mental health problems and the age at which these problems caused impairment in their life. Age of first impairment was taken as the main measure of age of onset. Finally, participants were categorised according to whether they had ever been admitted to a psychiatric unit (yes/no) based on data from CoMPaSS (number of admissions) and NCMH (ever admitted to hospital).

## Methods S4: Response rates by diagnosis

A total of 6920 participants were invited. The diagnoses of 16 of these participants were listed as unknown and are not included in the table below. Response rates include all participants who were invited and participated, regardless of whether they were later excluded. Response rates do not include individuals who were invited to participate in the pilot and validation study due to differences in the study protocol between the validation and main study phases (e.g. recruitment, time commitment, reimbursement).

Table S4 Response rates for participants sent invitation emails or letters broken down by diagnosis

|  | **Invited** | **Participated** | **Response rate** |
| --- | --- | --- | --- |
| **Healthy Controls** | 939 | 213 | 23% |
| **Unipolar Depressive Disorders^1^** | 1630 | 301 | 18% |
| **Bipolar Spectrum Disorders^2^** | 1115 | 158 | 14% |
| **Schizophrenia^3^** | 844 | 44 | 5% |
| **Co-morbid Anxiety and Depression^4^** | 532 | 139 | 26% |
| **Post-Traumatic Stress Disorder** | 476 | 65 | 14% |
| **Anxiety Disorders^5^** | 419 | 85 | 20% |
| **Other Psychotic Disorders^6^** | 245 | 18 | 5% |
| **Autism Spectrum Disorders^7^** | 172 | 32 | 19% |
| **Personality Disorders^8^** | 125 | 25 | 20% |
| **Other Psychiatric Disorders^9^** | 103 | 17 | 17% |
| **Attention Deficit Hyperactivity Disorder** | 88 | 11 | 13% |
| **Eating Disorders^10^** | 85 | 25 | 29% |
| **Obsessive Compulsive Disorder** | 32 | 9 | 28% |
| **Learning Difficulties and Disabilities^11^** | 34 | 0 | 0% |
| **Neurological Disorders^12^** | 27 | 1 | 4% |
| **Substance Use Disorders^13^** | 23 | 1 | 4% |
| **Other Mood Disorders^14^** | 15 | 8 | 53% |
| **Total** | 6904 | 1152 | 17% |

^1^Single episode or recurrent major depressive disorder, depressive disorder not otherwise specified (NOS) and post-natal depression; ^2^Bipolar disorder type I, type II, manic or hypomanic episode, cyclothymia and bipolar NOS; ^3^Schizophrenia and schizoaffective disorder – depressive type; ^4^Participants who reported co-morbid diagnoses of depression and anxiety with neither being the primary diagnosis; ^5^Panic disorder, agoraphobia, specific phobias, social phobias, generalised anxiety disorder, anxiety NOS; ^6^Schizoaffective disorder – bipolar type, schizoaffective disorder – NOS, delusional disorder, psychosis NOS, substance induced psychotic disorder, puerperal psychosis; ^7^Autism, Asperger’s syndrome; ^8^Borderline personality disorder, histrionic personality disorder and other personality disorders; ^9^This category includes invited participants with other behavioural or developmental disorders with onset in childhood or adolescence (such as oppositional defiant disorder), tic disorders or those rated as other psychiatric disorder, a category that includes participants that have subclinical symptoms of psychosis or mood disorder but have not been given a diagnosis, a history of self-harm without diagnosis or have experienced psychological distress (such as bereavement or work stress) requiring a period of leave or referral to occupational health or counselling services; ^10^Anorexia, bulimia, binge eating disorder and eating disorder NOS; ^11^Dyslexia, dyspraxia/developmental coordination disorder and intellectual disability; ^12^Huntington’s disease, Parkinson’s disease and dementia; ^13^Alcohol/other drugs abuse or dependence; ^14^Partipants rated as having a mood disorder that does not meet criteria for a specific diagnosis.

## Methods S5: Flow diagram of included and excluded participants


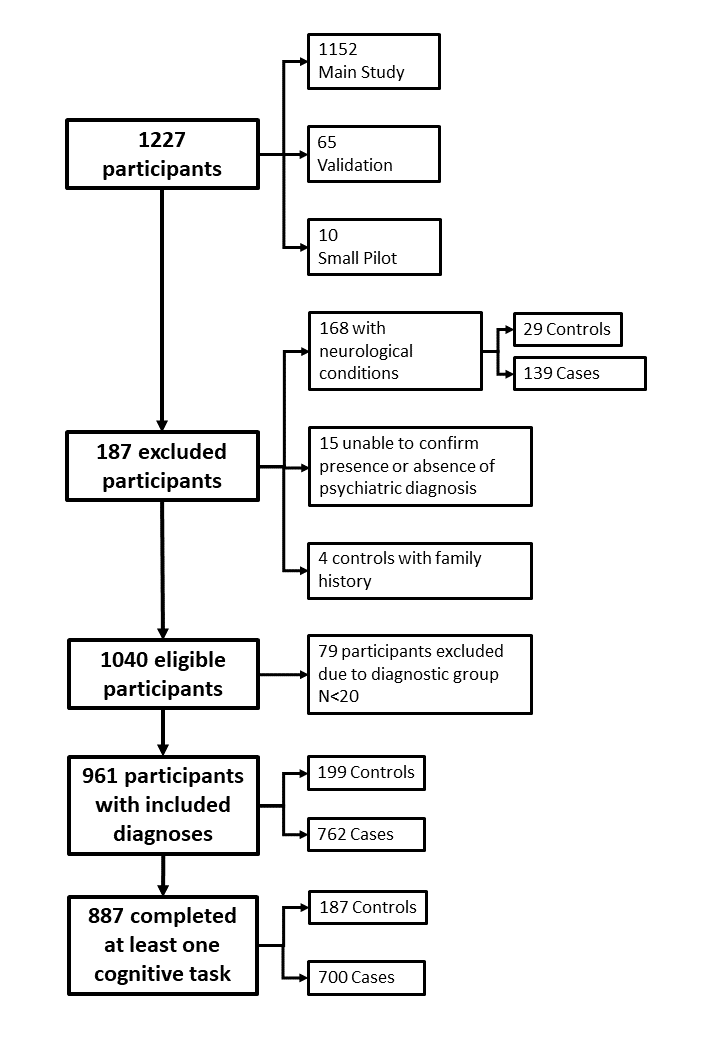


## Methods S6: Inclusion criteria for diagnostic comparisons

We sought to compare cognitive function in participants with major depressive disorder, bipolar disorder and schizophrenia. More conservative inclusion criteria were applied for these analyses given the self-report nature of the diagnoses for NCMH participants to ensure that the participants from this cohort were more comparable with those from CoMPaSS who were diagnosed using clinical interviews and case note review. The depression group included all participants who reported a diagnosis of major depressive disorder (single or recurrent episodes, with or without comorbid anxiety disorder) and had taken at least one antidepressant (N=295). The bipolar disorder group included all participants who reported a diagnosis of bipolar disorder (either mania or hypomania) and had taken at least one mood stabiliser or antipsychotic (N=116). The schizophrenia group included all participants who reported a diagnosis of schizophrenia or schizoaffective disorder – depressive type and had taken at least one antipsychotic (N=38).

# Supplementary Tables

## Table S1: Validation Study – Sample Characteristics

Table S1 Demographic and clinical characteristics of participants in the validation study

|  | **Healthy Controls** | **Major Depressive Disorder** | **Bipolar Disorder** | **Schizophrenia** |
| --- | --- | --- | --- | --- |
| **N** | 19 | 15 | 16 | 15 |
| **Age** | 49.8 (15) | 52.4 (10.5) | 51.1 (13.8) | 49.8 (12.4) |
| **Proportion Female (% Female)** | 12 / 19 (63%) | 10 / 15 (67%) | 11 / 16 (69%) | 5 / 15 (33%) |
| **Estimated IQ^1^** | 105.4 (17.8) | 100.2 (14.7) | 96.2 (19.6) | 86.9 (19.2) |
| **Highest Qualification** |  |  |  |  |
| None | 0 | 1 | 0 | 1 |
| 11+ | 0 | 0 | 0 | 1 |
| CSE or equivalent | 0 | 2 | 0 | 2 |
| GCSE or equivalent | 0 | 2 | 5 | 3 |
| A-level or equivalent | 5 | 5 | 5 | 6 |
| Degree | 8 | 3 | 5 | 1 |
| Postgraduate degree | 5 | 2 | 1 | 1 |
| **Lifetime Occupation** |  |  |  |  |
| Senior official | 0 | 0 | 1 | 1 |
| Professional | 8 | 7 | 5 | 1 |
| Technical | 3 | 1 | 3 | 2 |
| Administration | 2 | 4 | 2 | 1 |
| Service work | 3 | 2 | 1 | 5 |
| Trade work | 0 | 0 | 0 | 3 |
| Factory or plant work | 0 | 1 | 0 | 0 |
| Elementary occupation | 1 | 0 | 2 | 0 |
| Armed forces | 0 | 0 | 0 | 1 |
| Never worked | 0 | 0 | 1 | 0 |
| Full-time student | 1 | 0 | 0 | 0 |
| Voluntary work | 0 | 0 | 0 | 1 |
| **Proportion Taking Psychiatric Medication (% taking)** | 0/19 (0%) | 8 /15 (53%) | 14 /16 (88%) | 15/15 (100%) |
| **Current depression score^2^** | 2 (4) | 3 (6) | 4 (8) | 6 (5) |
| **Current anxiety score^2^** | 3 (6) | 8 (7) | 8 (5) | 12 (12) |
| **Current mania score^2^** | 5 (5) | 2 (5) | 2.5 (3) | 4 (4) |

Numbers indicate mean and standard deviation for continuous data and proportions for categorical data. Mood scores shown are based on Hospital Anxiety and Depression Scale (HADS) and Altman Mania Rating Scale (AMS) responses on the day participants completed the MCCB. ^1^IQ score estimated based on NART scores using the formulas described in [21]. ^2^Median and interquartile range are shown.

## Table S2: Performance on the MCCB and Online Battery

Table S2 Group performance on the MCCB and online battery

|  | **Schizophrenia** | | **Bipolar Disorder** | | **Major Depressive Disorder** | |
| --- | --- | --- | --- | --- | --- | --- |
|  | MCCB | Online | MCCB | Online | MCCB | Online |
| **Speed of Processing** | -1.04 (1.11) | -1.02 (1.13) | -0.75 (1.02) | -0.54 (1.12) | -0.49 (0.96) | -0.44 (0.77) |
| **Verbal Learning** | -1.35 (1.55) | -0.23 (1.28) | -0.48 (1.37) | 0.23 (1.35) | -0.36 (0.98) | -0.31 (1.46) |
| **Social Cognition** | -1.95 (1.04) | -0.99 (0.58) | -0.93 (0.68) | -0.27 (0.74) | -0.13 (1.23) | -0.24 (0.67) |
| **Working Memory** | -0.88 (1.04) | -0.47 (1.18) | -0.16 (0.88) | -0.72 (0.69) | -0.01 (1.07) | 0.59 (1.23) |
| **Visual Learning** | -0.63 (1.02) | -0.66 (0.99) | -0.09 (1.07) | -0.53 (0.94) | 0.04 (1.26) | -0.3 (0.84) |
| **Reasoning & Problem Solving** | -0.85 (1) | -0.79 (1.11) | -0.24 (1.19) | -0.44 (1.22) | -0.13 (1.27) | 0.13 (1.16) |
| **Attention** | -0.07 (0.96) | -0.85 (1.03) | -0.25 (1.31) | -0.55 (1.35) | 0.38 (1.2) | -0.26 (1.17) |
| **Vocabulary** | -1.04 (1.08) | -0.91 (1.14) | -0.52 (1.1) | -0.18 (1.01) | -0.29 (0.82) | 0.56 (0.66) |
| **Strategic Risk Taking** |  | -0.17 (1.07) |  | -0.04 (1.05) |  | 0.49 (0.49) |

Means and standard deviations for standardised scores (z-scores) are shown. Z-scores were calculated using the means and standard deviations of the control group. For the MCCB, only the Digit Symbol Coding is shown as a measure of speed of processing and only the Letter-Number Sequencing is shown as a measure of working memory. There is no equivalent measure to the Balloon Analogue Risk Task (strategic risk taking) in the MCCB.

## Table S3: Overall completion rates

Completion rates are shown for all participants who were included in the final sample and analyses. Tasks are shown in order of administration.

Table S3 Completion rates

| **Test** | **Digit Symbol Coding** | **Morphed Emotion Identification** | **Verbal Paired Associates** | **Backward Digit Span** | **Hartshorne Visual Working Memory** | **Matrix Reasoning** | **Balloon Analogue Risk Task** | **Multiple Object Tracking** | **Vocabulary** |
| --- | --- | --- | --- | --- | --- | --- | --- | --- | --- |
| **Healthy Controls** | 185 | 171 | 168 | 159 | 155 | 150 | 150 | 147 | 151 |
| **Bipolar Spectrum Disorders** | 143 | 131 | 127 | 125 | 119 | 117 | 115 | 106 | 109 |
| **Schizophrenia** | 43 | 42 | 41 | 41 | 40 | 40 | 39 | 40 | 39 |
| **Unipolar Depression** | 260 | 231 | 229 | 214 | 201 | 196 | 195 | 186 | 190 |
| **Anxiety Disorders** | 67 | 58 | 57 | 53 | 50 | 50 | 50 | 46 | 47 |
| **PTSD** | 48 | 38 | 38 | 37 | 35 | 33 | 35 | 32 | 33 |
| **Eating Disorders** | 21 | 18 | 18 | 16 | 15 | 16 | 16 | 14 | 15 |
| **Co-morbid Anxiety and Depression** | 113 | 97 | 96 | 92 | 88 | 85 | 85 | 83 | 84 |
| **Total** | **880** | **786** | **774** | **737** | **703** | **687** | **685** | **654** | **668** |

# Supplementary Figures

## Figure S1: Cognitive performance across domains


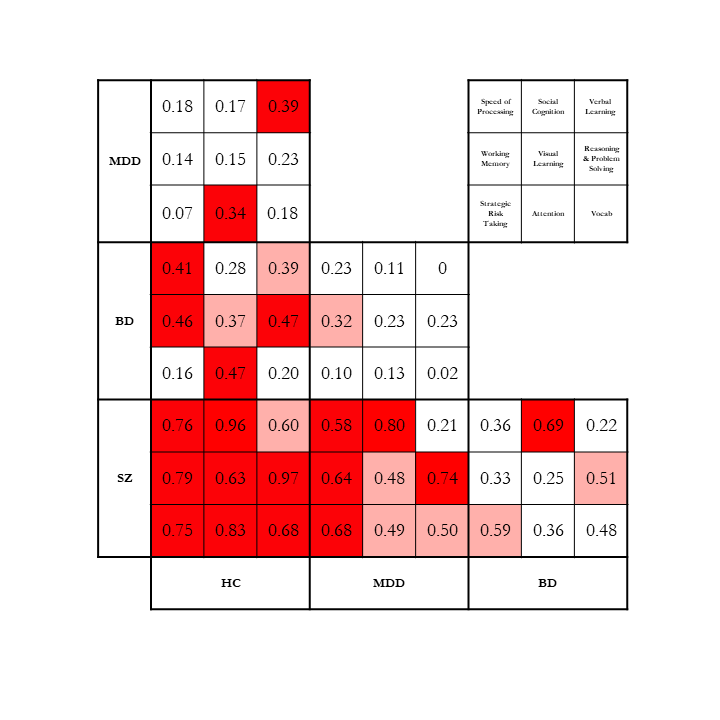


Figure S1 Pairwise comparisons Each 3x3 section displays the Hedges’ g effect sizes for the differences between two groups for each domain of cognition. Positive effect sizes indicate that the group on the horizontal bottom row performed better than the group on the left-hand vertical column. Lighter shade *P*<.05, darker shade *P*<.005 (threshold using Bonferroni-correction for 9 domains plus ‘g’).
